# Radiofrequency Ablation of a Left Atrial Appendage Tachycardia on ECMO Support

**DOI:** 10.1155/2013/203241

**Published:** 2013-11-28

**Authors:** Mohsin Khan, Andre Gauri, Ronald Grifka, Darryl Elmouchi

**Affiliations:** ^1^Department of Internal Medicine, Spectrum Health Medical Group, 100 Michigan ST NE, MC 127, Grand Rapids, MI 49503, USA; ^2^College of Human Medicine, Michigan State University, Grand Rapids, MI 49503, USA; ^3^Frederik Meijer Heart & Vascular Institute, Spectrum Health, Grand Rapids, MI 49503, USA; ^4^Department of Pediatrics, University of Michigan School of Medicine, Ann Arbor, MI 48109-5718, USA

## Abstract

Extracorporeal membrane oxygenation (ECMO) has been utilized in the pediatric population for cardiogenic shock secondary to medically intractable arrhythmias. There is limited experience with cardiac radiofrequency ablation (RFA) on these patients while on ECMO. A 7-year-old girl presented with a tachycardia-mediated cardiomyopathy secondary to a left atrial appendage tachycardia. She suffered a cardiac arrest due to pulseless electrical activity and was placed on ECMO. Due to elevated left atrial pressures and the refractoriness of her arrhythmia to cardioversion and antiarrhythmic therapy, while on ECMO, blade atrial septostomy and radiofrequency ablation were performed. The patient tolerated the procedure well and was successfully decannulated. Her cardiac function normalized within four weeks of the ablation procedure. Twelve months after the procedure, she remains completely well, with no symptoms or tachycardia.

## 1. Introduction

The left atrial appendage (LAA) is an uncommon site for origin of ectopic atrial tachycardia [1]. It often presents as incessant tachycardia not responsive to adenosine. Tachycardia mediated cardiomyopathy can result as well. Radiofrequency ablation is the treatment of choice and is considered safe and effective [2, 3]. In the presence of cardiogenic shock secondary to medically intractable arrhythmias, extracorporeal membrane oxygenation (ECMO) support may be considered. This is seen most often in pediatric patients who develop these arrhythmias after cardiac surgery for congenital heart disease [4]. While in most of these cases, ECMO support is utilized while awaiting recovery of cardiac rhythm, there have been very few cases reported when radiofrequency ablation was performed for intractable arrhythmias while on ECMO support [5]. We present an unusual case of an otherwise healthy 7-year-old girl who presented with an ectopic atrial tachycardia arising from the left atrial appendage and underwent radiofrequency ablation on ECMO support.

## 2. Case Report

A previously healthy 7-year-old girl had a three-day history of cough and flu-like symptoms. Her grandfather noted she had a fast heart rate. She was taken to a local hospital reporting increasing fatigue and dyspnea with exertion for the prior three weeks. The patient was hemodynamically stable at the time of presentation. Her heart rate was 230 beats per minute. Adenosine was given multiple times which resulted in transient AV dissociation, but had no effect on the tachyarrhythmia. She received a bolus of procainamide, and was transferred to our children's hospital for further evaluation and treatment.

**Figure 1:**
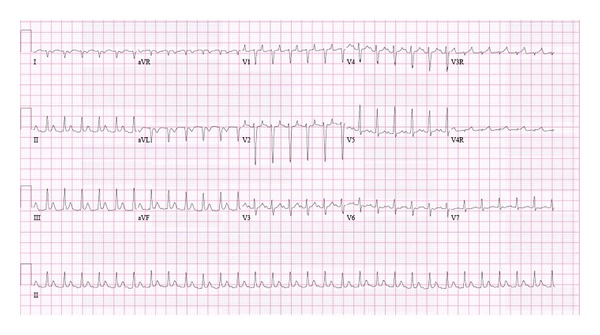
ECG showing narrow complex tachycardia with a long RP pattern.

Upon arrival, her heart rate was 175 beats per minute. The remainder of her vital signs were normal, as was her physical examination. The initial ECG demonstrated a narrow complex tachycardia (Figure 1). An echocardiogram revealed a dilated left ventricle with globally depressed systolic function (shortening fraction [SF] = 18%), and moderate mitral regurgitation. NT-Pro BNP (brain natriuretic peptide) was elevated (5599, normal range 5–1800 ng/L), and serial troponin levels were undetectable. Tachycardia-mediated cardiomyopathy was suspected. The procainamide infusion was continued, and the electrophysiology service was consulted for possible electrophysiological (EP) study. Intravenous and oral metoprolol tartrate were administered. Despite the procainamide infusion and beta blockade, she continued to remain tachycardic (heart rate 140 to 180 beats per minute).

She was taken to the catheterization lab for an electrophysiology study. She was intubated and placed under general anesthesia by a pediatric anesthesiologist. Three femoral venous sheaths were inserted into the right femoral vein via the modified Seldinger technique. A decapolar coronary sinus catheter (Biosense Webster, Diamond Bar, CA, USA) was placed. A D-curve 4.0 mm Navistar ablation and mapping catheter (Biosense Webster, Diamond Bar, CA, USA) was inserted to the His position. A quadripolar catheter (St. Jude Medical, St. Paul, MN, USA) was placed in the right ventricular apex. The tachycardia cycle length = 330 msec with 1 : 1 conduction. The PR interval length = 130 msec, QRS duration = 77 msec, AH interval = 70 msec, and the HV interval = 40 msec. A three-dimensional electro-anatomic activation map (CARTO 3, Biosense Webster, Diamond Bar, CA, USA) of the right atrium displayed a broad septal region of early activation. The activation in the coronary sinus was distal to proximal, consistent with a left atrial focus.

While performing the mapping procedure, before transseptal access or ablation was attempted, the blood pressure dropped precipitously. On fluoroscopy, the cardiac silhouette demonstrated no cardiac motion, consistent with pulseless electrical activity (PEA). Cardiopulmonary resuscitation (CPR) was initiated and intravenous epinephrine was given with return of circulation. An emergent transthoracic echocardiogram revealed no pericardial effusion. Over the next 120 minutes, the patient suffered three further PEA arrests, each time with return of spontaneous circulation after CPR and epinephrine. Due to hemodynamic instability and poor left ventricle contractility, we initiated emergent veno-arterial extracorporeal membrane oxygenation (ECMO) utilizing the right femoral vein and right carotid artery. Using ECMO support, her condition stabilized promptly. However, she remained in atrial tachycardia with 1 : 1 conduction in spite of repeated cardioversions and intravenous amiodarone. An echocardiogram showed severely depressed left ventricular function (SF = 6%) and mild left atrial enlargement. The next day, the echocardiogram revealed increasing left atrial size and pressure. An atrial septostomy was indicated to relieve left atrial hypertension. Given the incessant nature of her tachycardia and failure to resolve on antiarrhythmic medication, a left atrial ablation would be performed following the atrial septostomy procedure.

**Figure 2:**
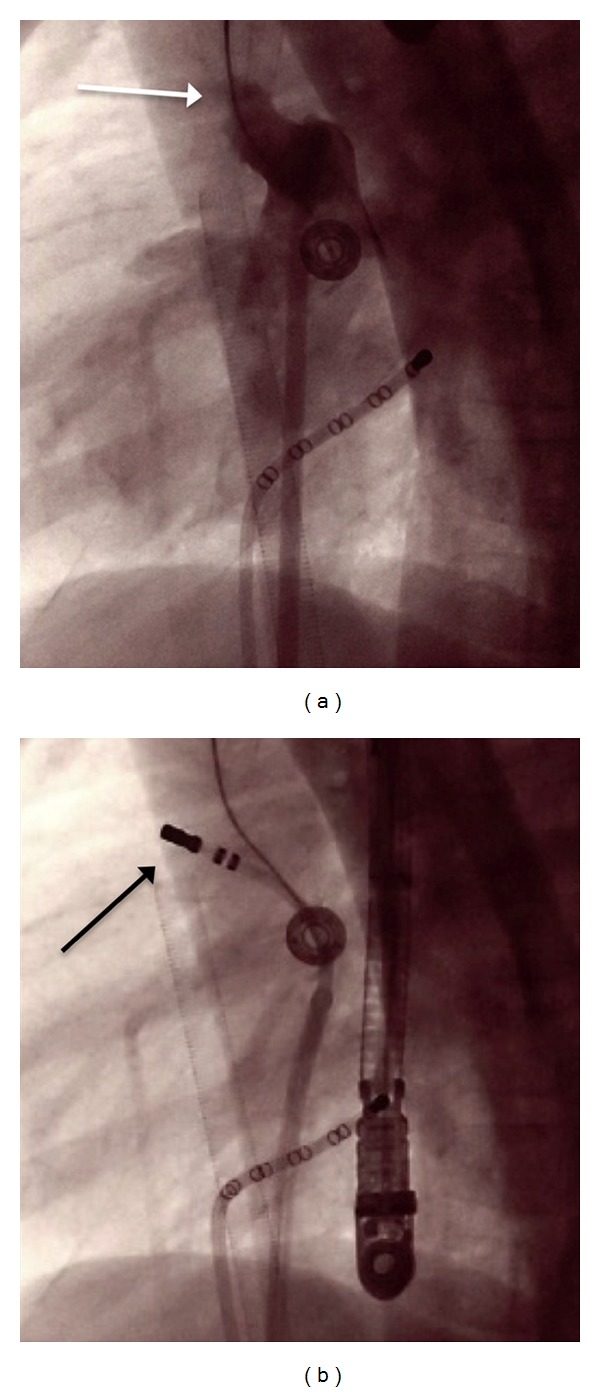
LAO 90 degree contrast injection of LAA ((a) marked by white arrow) and successful ablation site (black arrow in (b)).

**Figure 3:**
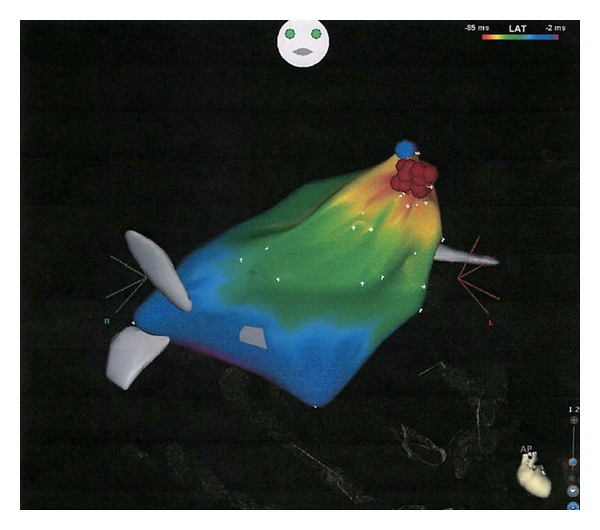
3D activation map of left atrium in AP projection.

The patient was bought to the catheterization lab on ECMO support. A transseptal puncture was performed using standard technique, followed by a blade atrial septostomy using a 13.4 mm blade catheter (Cook Inc, Bloomingtion, IN, USA). With access to the left atrium obtained, a detailed electro-anatomic map of the left atrium was created with the CARTO mapping system. The tachycardia cycle length was now 290 msec. The electro-anatomic map clearly demonstrated focal activation originating in the very distal left atrial appendage. A contrast injection into the left atrium demonstrated a long and trabeculated left atrial appendage (Figure 2). Detailed activation mapping within the left atrial appendage found sites that were very distal and anterior, 60 ms presystolic to the *P* wave (Figure 3). Radiofrequency ablation was performed using 20 watts, titrating up to 30 watts, which resulted in termination of the tachycardia. The tachycardia terminated for 15 minutes, but then recurred at a cycle length = 320 msec. Additional ablation was performed using 35 watts, with the ablation catheter placed at the distal tip of the left atrial appendage. This lesion caused the tachycardia to terminate within one second of energy delivery. Three 60-second lesions were performed in this region using 35 watts. Following these three ablation lesions in the distal atrial appendage, the tachycardia did not return. Aggressive rapid atrial pacing was performed, including during an isoproterenol infusion titrated up to 3 mcg/minute, and no tachycardia was induced. Immediately upon return of sinus rhythm, a transesophageal echocardiogram demonstrated improved left ventricular systolic function. She was transferred back to the pediatric intensive care unit on ECMO. The arterial catheter waveform displayed pulsatile flow and her ECMO requirements reduced substantially. She made steady progress and was easily weaned from ECMO support 40 hours after the ablation procedure.

Post-ECMO decannulation, a repeat echocardiogram demonstrated progressive improvement of the left ventricular systolic function. As the sedation was weaned, she showed remarkable improvement in her neurological status including purposeful movements. Three days after the procedure, she was extubated uneventfully and displayed no neurological deficits. With continued excellent progress, she was discharged to home 10 days after the procedure. Follow-up clinic evaluations demonstrated normalization in the left ventricle systolic function (SF = 32%) and return to her baseline activity level. For 12 months after the procedure, she has not had any atrial tachycardia noted by report, exam, or repeated 14 day continuous ambulatory monitoring exam. She has made a full recovery and is now on a local soccer team.

## 3. Discussion

Focal atrial tachycardia can arise from various portions of the atrium [6]. These include the crista terminalis [7], around or inside the coronary sinus [8], near or inside the pulmonary veins, superior vena cava, atrial septum, and Koch's triangle. Left atrial appendage tachycardia, is uncommon, accounting for 3% of all ectopic atrial tachycardias. The left atrial appendage is the remnant of the embryonic left atrium and is located between the mitral valve and left upper pulmonary vein. The tachycardia focus tends to be localized to the base of the atrial appendage, primarily on the medial side [9]. Often, it is resistant to termination with adenosine. Radiofrequency ablation is the treatment of choice, and is associated with a high success rate. The patient's initial presentation was due to cardiomyopathy symptoms secondary to persistent tachycardia. Due to marginal cardiac output and diminished cardiac reserve, the patient was unable to tolerate general anesthesia, and suffered several PEA arrests. Several studies have shown that early use of ECMO support has been associated with improved outcomes, and we believe the decision to promptly utilize ECMO was life saving in our patient. While there is data supporting the use of ECMO to provide cardiac support in pediatric patients in low output states due to arrhythmias, there is very little data reporting the safety and efficacy of performing radiofrequency ablation during ECMO support. Dyamenahalli et al. described the experience of ECMO support for intractable primary arrhythmias in newborns and infants [5]. Out of nine patients on ECMO support, two underwent radiofrequency ablation. All patients survived to hospital discharge, and there was one late death on follow-up for 5 years. A retrospective study by Booth et al. reported cardiac catheterization procedures on pediatric patients on ECMO support [10]. Sixty catheterizations were performed on 54 pediatric patients, only three catheterizations were for arrhythmia ablation, and there was a low risk of catheterization-related complications with the use of ECMO support. There is one report of a 24-year-old postpartum patient who underwent successful radiofrequency ablation for atrial tachycardia while on ECMO support [11]. The successful outcome from our case suggests that radiofrequency ablation for ectopic atrial tachycardia, even in the distal left atrial appendage, can be performed safely on young patients while on ECMO support, and can be life saving, as demonstrated by this case.
